# Supplementary material for: Time-compressed preplay of anticipated events in human primary visual cortex
Source: Nat Commun. 2017 May 23;8:15276. doi: 10.1038/ncomms15276 (PMC5457495; doi:10.1038/ncomms15276)
Supplement: Supplementary Information — Supplementary Figures [file ncomms15276-s1.pdf]

Supplementary Figures

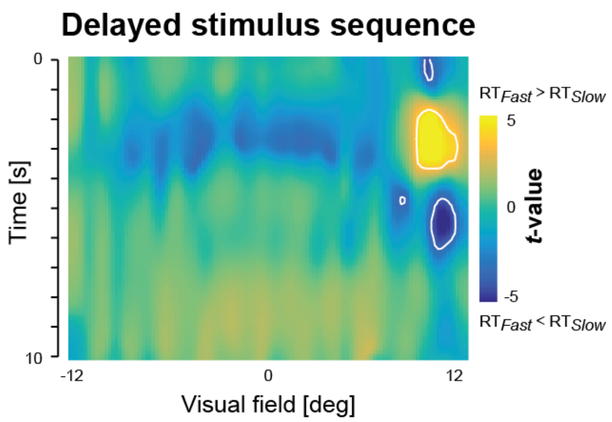

Supplementary Figure 1. Contrast of BOLD responses for fast compared to slow detection trials show BOLD differences specific to the retinotopic location of the last stimulus. Fitted BOLD responses for fast and slow detection trials were compared using a paired-sample *t*-test. The white areas highlight locations for which BOLD amplitude differs significantly between fast and slow detection trials ($P < 0.01$, uncorrected). The resulting statistical *t*-map is smoothed for visualization purposes.

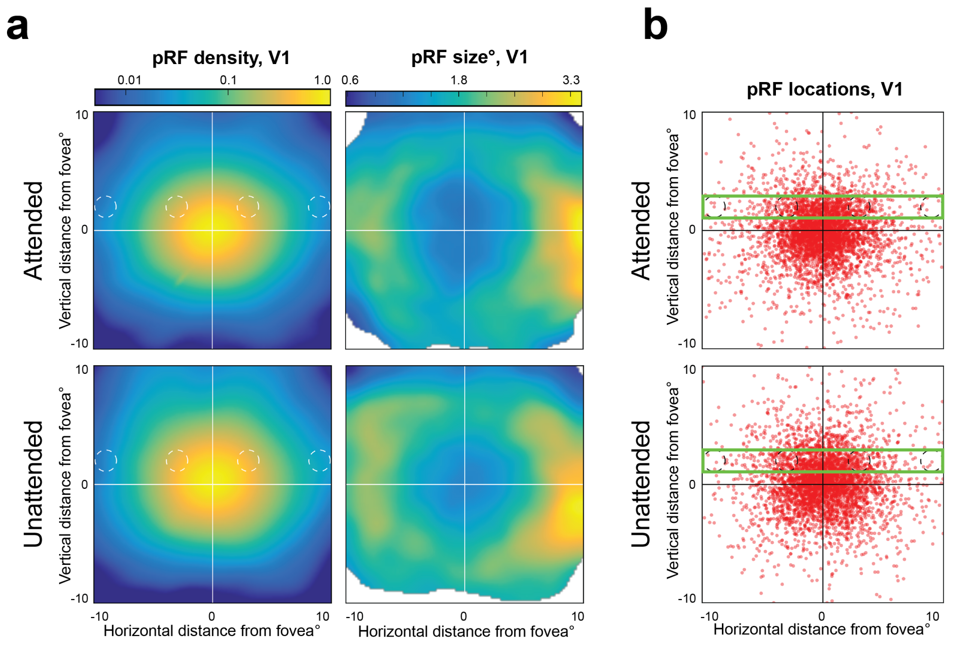

Supplementary Figure 2. pRF properties and voxel selection. (**a**) pRF density and pRF size in V1, for the Attended (*top*) and Unattended Condition (*bottom*) averaged across subjects (N=29). (**b**) pRF locations in V1 (red). Data is pooled across subjects. The green box depicts the voxels along the stimulus path (y=1.4° to y=2.6°, and x=-10° to x=10°) that were selected for the analysis.

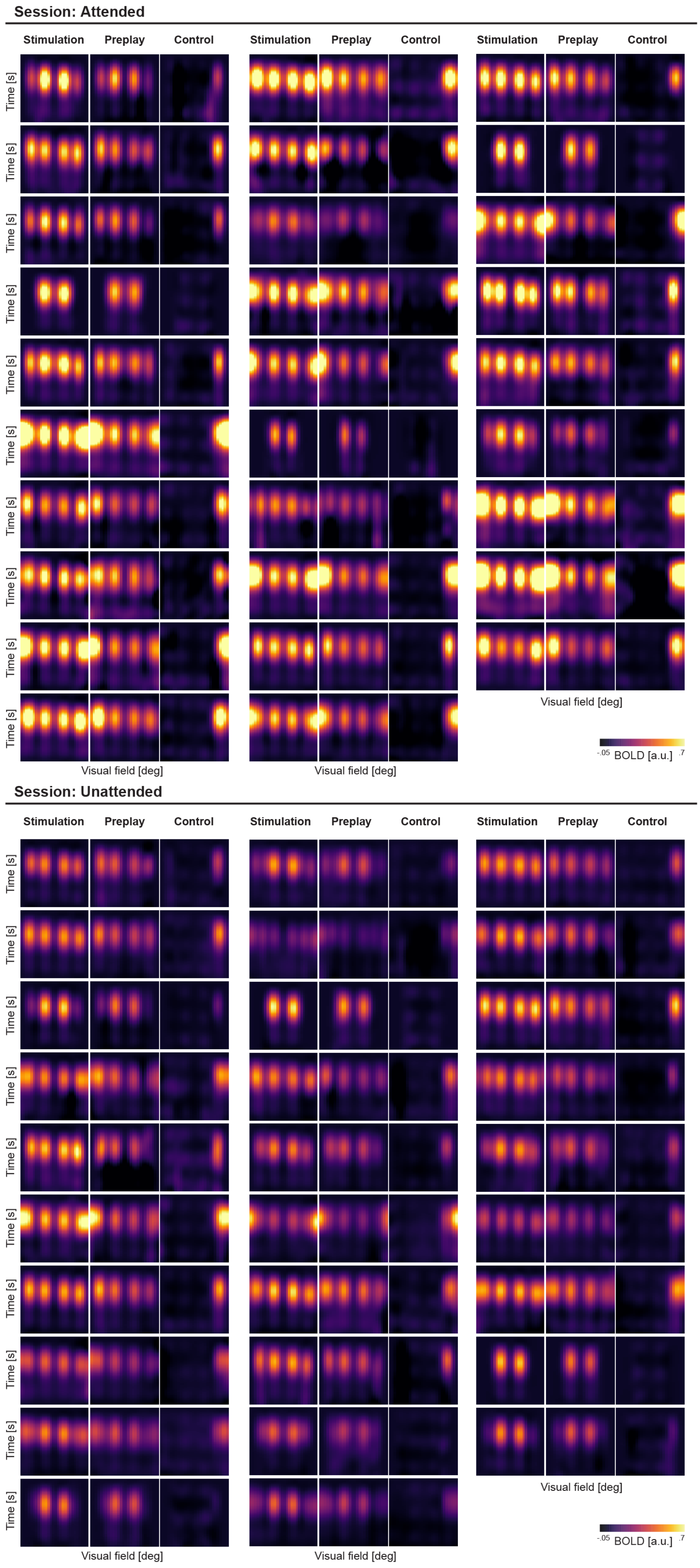

Session: Attended
Stimulation
Preplay
Control
Stimulation
Preplay
Control
Stimulation
Preplay
Control
Time [s]
Visual field [deg]
Visual field [deg]
Visual field [deg]
-.05 BOLD [a.u.] .7
Session: Unattended
Stimulation
Preplay
Control
Stimulation
Preplay
Control
Stimulation
Preplay
Control
Time [s]
Visual field [deg]
Visual field [deg]
Visual field [deg]
-.05 BOLD [a.u.] .7

Supplementary Figure 3. Fitted BOLD responses for individual participants as a function of retinotopic horizontal eccentricity during presentation of the stimulus sequence (*left column*), preplay (*middle column*) and no preplay control (*right column*) for the Attended (*top*) and Unattended (*bottom*) Condition, respectively. Averaged for Left-to-right and Right-to-left blocks.

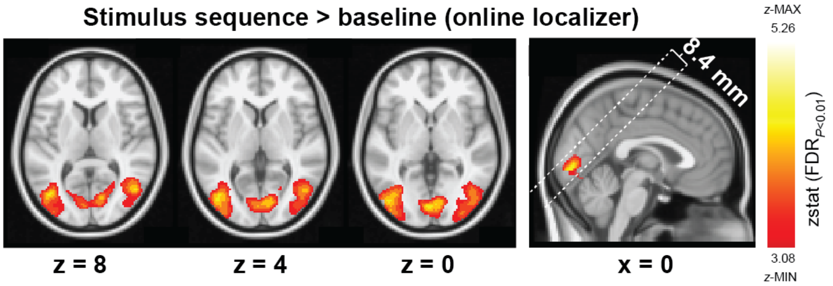

Supplementary Figure 4. Results of online localizer to aid slice positioning of the ultra-fast scanning sequence. GLM *z*-statistic contrasting stimulus sequence compared to baseline, superimposed on a standard brain template; averaged for the Attended and Unattended Condition (*left*). Dashed white lines highlight the approximate slices thickness and coverage of the voxels active during the online localizer (*right*). Across subjects, the 2 slices covered 64% (28% SD) of the activated voxels in V1 and 31% (12% SD) in hMT+; respectively averaged over Attended and Unattended Condition.

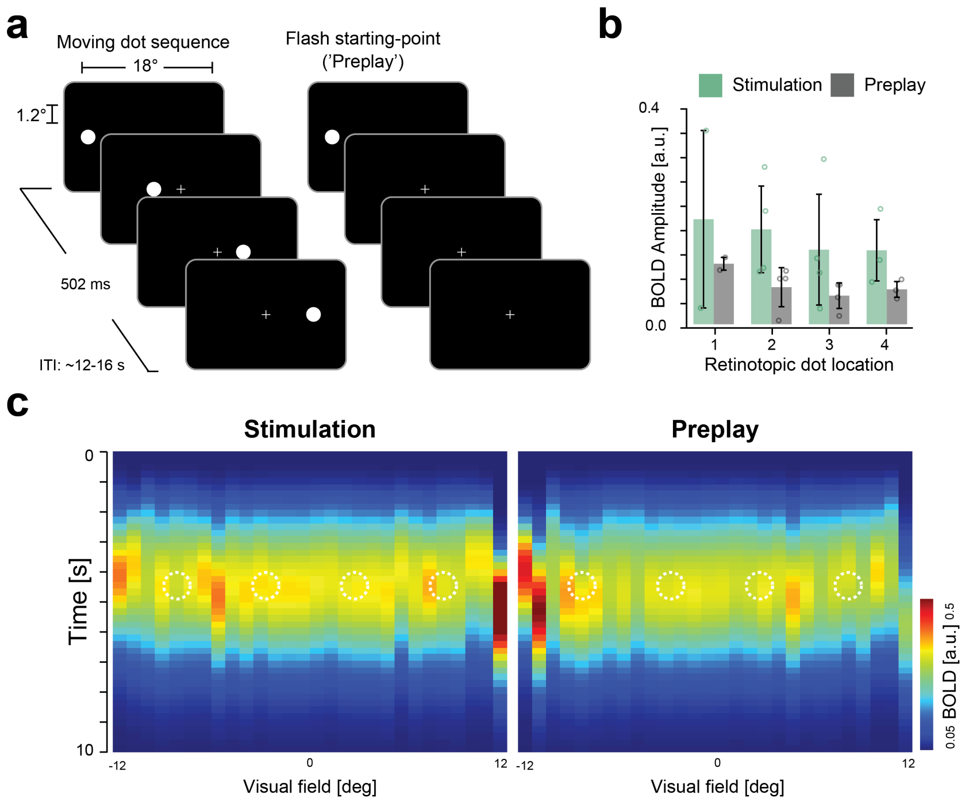

Supplementary Figure 5. Control experiment (N=4) with variable ITI (~12-16 s). (**a**) Experimental paradigm. Participants were instructed to detect a dimming of the fixation cross (reduction of stimulus contrast by 30%). Notably, here the path of the dot sequence crosses fixation. (**b**) Corresponding BOLD amplitudes at the stimulus locations for the Stimulation (green) and Preplay (grey) Condition. (**c**) Fitted BOLD responses as a function of retinotopic horizontal eccentricity during presentation of the stimulus sequence (*left*) and Preplay (*right*). Dashed circles depict horizontal stimulus locations. Error bars denote $\pm$ s.d.

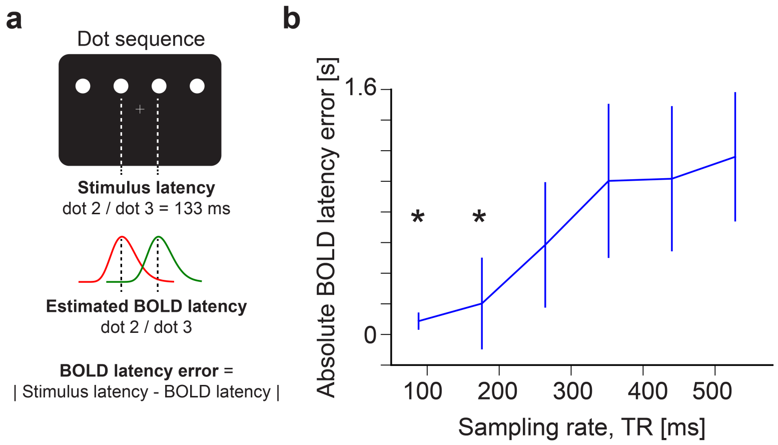

Supplementary Figure 6. Detection of BOLD latency differences depends on fast fMRI sampling rate. (**a**) Existing fMRI data (TR = 88 ms) were down-sampled to illustrate the dependency of detecting relatively short stimulus latencies on sampling rate. (**a**) The BOLD latency error (closer to zero is better) was calculated as the absolute difference between the known stimulus latency (133 ms) and the estimated BOLD latency for two dot stimuli during the presentation of the stimulus sequence. (**b**) BOLD latency error increases with sampling rate. Statistical significance is based on one-sample *t*-tests comparing the estimated BOLD latency between the two stimuli across participants against zero. In the tested range, only sampling rates < 200 ms allowed for an accurate estimation of the known stimulus latency. Error bars denote ± s.d.; * = $P<0.05$.

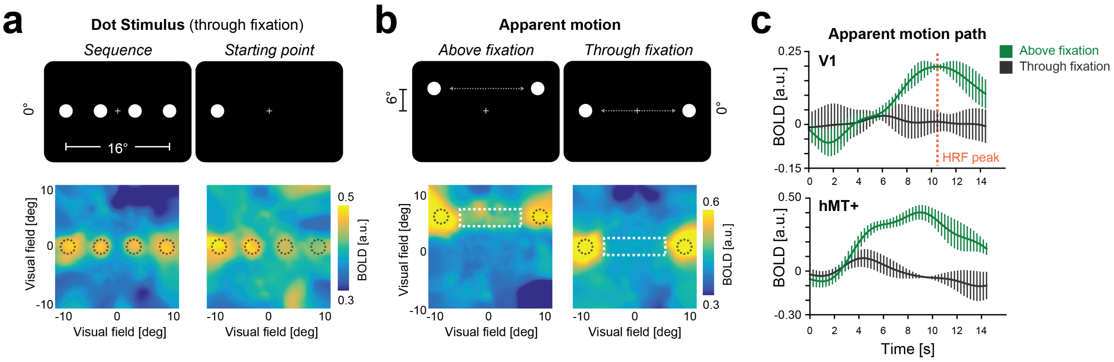

Supplementary Figure 7. Control analysis (N=4) contrasting our results with an apparent motion paradigm. (**a**) Stimulus sequence and preplay through fixation (*top*). Group average of the pRF-based reconstruction (see Materials and Methods) from V1 BOLD activity (*bottom*). (**b**) Apparent motion paradigm above fixation and through fixation (*top*). Group average of the pRF-based stimulus reconstruction from V1 BOLD activity. (**c**) BOLD time-courses along the apparent motion path (white box) for V1 (*top*) and hMT+ (*bottom*) for the apparent motion paradigm. The group averaged V1 BOLD response shows a peak at around 10 s after stimulus onset. Dashed circle highlights the position of the respective stimulus positions. Error bars denote ± s.e.m.

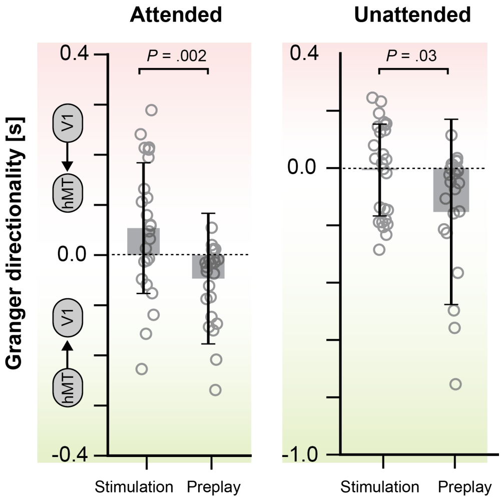

Supplementary Figure 8. Probing the directionality of the hMT+, V1 correlation using Granger correlation (GC) analysis. Within the GC framework, a directionality value < 0 means that hMT+ is 'driving' V1 (*green shaded area*), a directionality value > 0 suggests the opposite direction, i.e., that V1 is 'driving' hMT+ (*red shaded area*). The results show that hMT+ is 'driving' V1 in the Preplay Condition (Attended: $t(23) = -3.50$; $P = 0.002$; Unattended: $t(25) = -2.68$; $P = 0.013$), but not in the Stimulation Condition (Attended: $t(23) = 1.97$; $P = 0.059$; Unattended: $t(25) = -0.22$; $P = 0.829$). The difference of the granger directionality between Stimulation and Preplay Condition was tested using a two-sided two-sample *t*-test (Attended: $t(23) = 3.38$; $P = 0.002$; Unattended: $t(25) = -2.26$; $P = 0.033$).
